# Falciparum Malaria-Induced Splenic Infarction

**DOI:** 10.1590/0037-8682-0330-2023

**Published:** 2023-09-22

**Authors:** Ramazan Orkun Önder, Abdulkadir Kaya, Serdar Aslan

**Affiliations:** 1 Giresun University, Faculty of Medicine, Department of Radiology, Giresun, Turkey.

**FIGURE 1:**
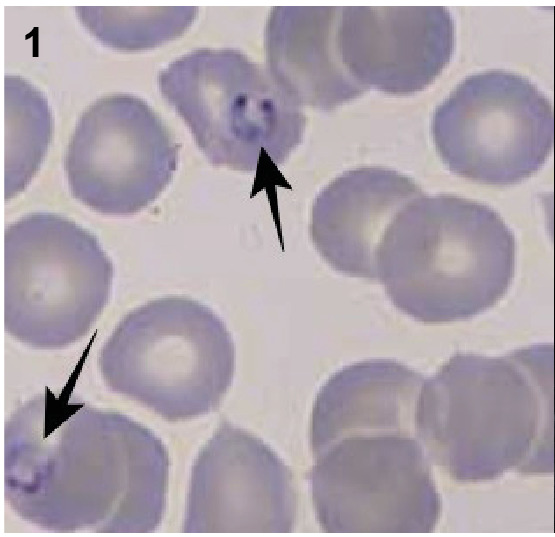
Appearance of banana-shaped *Plasmodium falciparum* gametocytes in erythrocytes on peripheral smear (black arrows).

**FIGURE 2:**
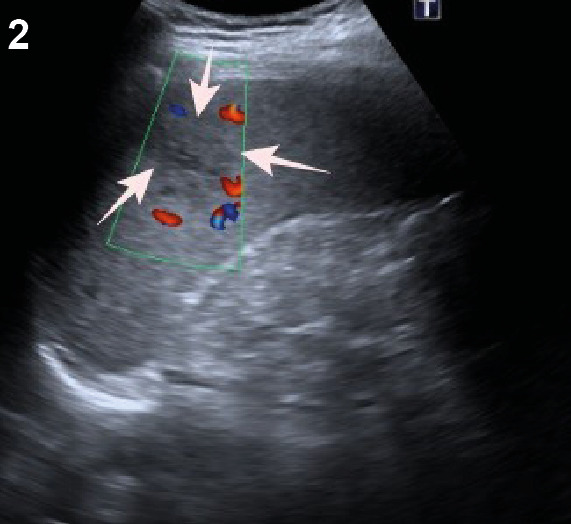
Ultrasonography showing a patchy hypoechoic area in the spleen (white arrows).

**FIGURE 3:**
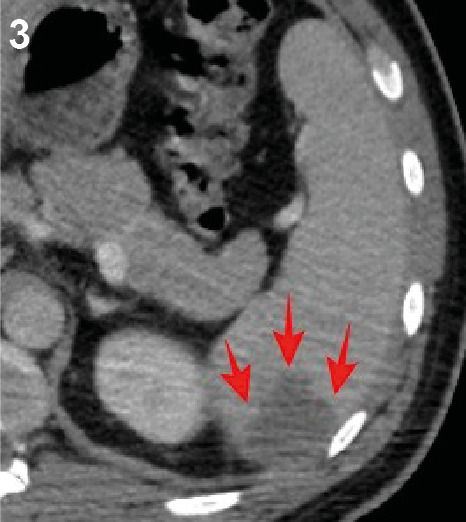
Venous phase contrast-enhanced computed tomography showing the infarct area in the spleen (red arrows).

On the final day of a 3-day oral artemether-lumefantrine combined malaria treatment, a 30-year-old man in the infectious diseases ward began experiencing pain in the left hypochondriac region. A peripheral smear examination revealed the presence of banana-shaped *Plasmodium falciparum* gametocytes within erythrocytes (Figure 1). Additionally, a rapid diagnostic immunoassay confirmed the presence of *P. falciparum* antigen in the patient’s blood. Abdominal ultrasonography identified a patchy hypoechoic lesion in the spleen (Figure 2), which was later confirmed as an infarct area through venous phase contrast-enhanced abdominal computed tomography (Figure 3). Observations also included splenomegaly, with a vertical length of 146 mm, and an increased splenic vein diameter of 11 mm. These findings were attributed to a splenic infarction caused by malaria. Malaria can lead to various splenic complications, including splenic infarction, spontaneous splenic rupture, hyperreactive malarial syndrome, hypersplenism, ectopic spleen and splenic torsion, and splenic cysts^1^. Splenic infarction, although not commonly observed, is likely underdiagnosed in many instances of complicated malaria^2^ ^,^ ^3^. Despite its rarity, splenic infarction should be considered as a potential complication in patients experiencing left quadrant pain during malaria treatment.
